# The Roles of Family B and D DNA Polymerases in *Thermococcus* Species 9°N Okazaki Fragment Maturation*

**DOI:** 10.1074/jbc.M115.638130

**Published:** 2015-03-26

**Authors:** Lucia Greenough, Zvi Kelman, Andrew F. Gardner

**Affiliations:** From ‡ New England Biolabs, Inc., Ipswich, Massachusetts 01938 and; the § National Institute of Standards and Technology, Rockville, Maryland 20850

**Keywords:** DNA, DNA enzyme, DNA polymerase, DNA replication, DNA synthesis, nucleic acid enzymology, Okazaki fragment, lagging strand, replisome

## Abstract

During replication, Okazaki fragment maturation is a fundamental process that joins discontinuously synthesized DNA fragments into a contiguous lagging strand. Efficient maturation prevents repeat sequence expansions, small duplications, and generation of double-stranded DNA breaks. To address the components required for the process in *Thermococcus*, Okazaki fragment maturation was reconstituted *in vitro* using purified proteins from *Thermococcus* species 9°N or cell extracts. A dual color fluorescence assay was developed to monitor reaction substrates, intermediates, and products. DNA polymerase D (polD) was proposed to function as the replicative polymerase in *Thermococcus* replicating both the leading and the lagging strands. It is shown here, however, that it stops before the previous Okazaki fragments, failing to rapidly process them. Instead, Family B DNA polymerase (polB) was observed to rapidly fill the gaps left by polD and displaces the downstream Okazaki fragment to create a flap structure. This flap structure was cleaved by flap endonuclease 1 (Fen1) and the resultant nick was ligated by DNA ligase to form a mature lagging strand. The similarities to both bacterial and eukaryotic systems and evolutionary implications of archaeal Okazaki fragment maturation are discussed.

## Teaser

**Background:** During replication, the lagging strand is synthesized discontinuously from a series of Okazaki fragments.

**Results:** Okazaki fragment maturation was reconstituted using purified proteins from *Thermococcus.*

**Conclusion:** In *Thermococcus*, efficient Okazaki fragment processing requires DNA polymerase B, flap endonuclease, and DNA ligase.

**Significance:** Okazaki fragment maturation in *Thermococcus* shares similarities to both bacterial and eukaryotic systems.

## Introduction

DNA replication is a conserved process throughout all domains of life (1). Due to the antiparallel nature of double-stranded DNA and the uni-directionality of DNA polymerases, the leading strand replicates continuously, whereas the lagging strand is synthesized discontinuously from a series of Okazaki fragments. The Okazaki fragment on the lagging strand starts as a short RNA primer synthesized by the primase. Then the processivity factor (the sliding clamp) assembles around the primer and binds DNA polymerase. The DNA polymerase-sliding clamp extends the RNA primer to synthesize the complementary strand. To form an uninterrupted lagging strand, RNA primers are removed, the gap in the DNA is filled and the Okazaki fragments are joined together.

Each domain of life accomplishes this objective using different mechanisms and components. In bacteria, the lagging strand is synthesized by DNA polymerase III holoenzyme (pol III) whereas DNA polymerase I (pol I) is the major polymerase that carries out Okazaki fragment maturation. pol I uses its polymerase activity to extend nicks or gaps left by pol III and its 5′-3′ exonuclease activity to degrade the downstream RNA primer. The nick is sealed by DNA ligase. In Eukarya, the same requirements are fulfilled using a different repertoire of enzymes. The lagging strand polymerase, pol δ, synthesizes the lagging strand and displaces the RNA primers into a flap structure. Flap endonuclease (Fen1) removes the flap and the nick is sealed by DNA ligase to generate a continuous double-stranded DNA (2).

Lagging strand synthesis and Okazaki fragment maturation are not as well understood in Archaea. The majority of characterized archaeal species (excluding the known Crenarchaea) encode both members of Family B DNA polymerase (polB) as well as the archaeal specific Family D DNA polymerase (polD) (3). Several lines of evidence suggest that polD is the main replicative polymerase for both the leading and lagging strand synthesis. In some species, polD is the only essential DNA polymerase for cell viability, whereas in others, both polB and polD are required (4–6). polD forms complexes with several key replisome proteins *in vivo*, whereas polB does not (7). In addition, the ability of polD to efficiently extend an RNA primer fulfills a requirement for both a leading and lagging strand DNA polymerase (8, 9). Thus, polD was suggested to replicate at least the lagging strand and likely the leading strand as well (10). In this study, the roles of polD and polB during Okazaki fragment maturation were evaluated using *in vitro* assays with proteins purified from *Thermococcus* species 9°N or cell extracts. The data suggest that Okazaki fragment maturation is a hybrid of the bacterial and eukaryal systems.

## EXPERIMENTAL PROCEDURES

### 

#### 

##### Enzymes and Reagents

All restriction endonucleases, modifying enzymes, polB (9°N_m_ DNA polymerase; 9°N/E143D), 9°N DNA ligase, T4 DNA ligase, nucleotides, and single-stranded M13mp18 DNA (ssM13) were from New England Biolabs, Inc. (Ipswich, MA). 9°N polD was purified as previously described (8). A DNA extension primer (5′-TAM-CGC CAG GGT TTT CCC AGT CAC GAC), labeled with 5-carboxytetramethylrhodamine (TAM)^2^ and a RNA-DNA blocking oligonucleotide (5′-rGrCrC rArArG rCrUrU rGCA TGC CTG CAG GTC GAC TCT AGA GGA TCC CCG GGT ACC GAG CTC GAA TT-FAM-3′) 3′-labeled with 5-carboxyfluorescein (FAM) were purchased from Integrated DNA Technologies (IDT, Coralville, IA). Aphidicolin was from Sigma.

##### Strains

*Thermococcus kodakarensis* (Tko) KW128 (wild-type (Tko)) strain was grown as previously described (5). Tko cell extracts were prepared by suspending cell paste (1 g) in 1 ml of Buffer A (20 mm Tris-HCl, pH 7.5, 50 mm NaCl). Suspended cells were sonicated for 3 min and centrifuged for 10 min at 13,000 rpm to remove cell debris. The extract supernatant was mixed with glycerol to 50% final concentration, and stored at −20 °C.

##### Immunodepletion of polB or polD from Tko Extracts

*Thermococcus* species 9°N lacks tools to construct gene deletions or genetic screens to study replication. Instead, immunodepletion of polB or polD from Tko cell extracts was used to probe function. polB- or polD-immunodepleted Tko cell extracts were prepared as follows. Tko cell extracts were diluted to a final concentration of 1 mg/ml in Buffer A and pre-cleared with protein G magnetic beads according to the manufacturer (New England Biolabs). Then 200 μl of pre-cleared Tko cell extract was incubated with 10 μl of dH_2_O, guinea pig anti-polD, or anti-polB antibodies (5) with gentle rocking at 4 °C for 16 h. Then 50 μl of protein G magnetic beads were added to capture the antibody-protein complex. The mixture was incubated for an additional 3 h at 4 °C and a magnetic field was applied to remove bead complexes. The immunodepleted supernatant was removed and stored.

Western blots were performed to assess polB and polD immunodepletion. Tko extracts or immunodepleted extracts were separated by electrophoresis, transferred onto membranes, and probed by incubation with rabbit polyclonal antisera raised against either 9°N polB (anti-polB) or 9°N polD (anti-polD).

##### Dual Label Fluorescence Okazaki Maturation Assay

A dual-label fluorescence assay was designed to mimic Okazaki fragment maturation conditions *in vivo*. The Okazaki fragment maturation assay detects two fluorescent dyes simultaneously and allows monitoring of DNA polymerase synthesis from the 5′-TAM-labeled extension primer and Fen1 and DNA ligase processing of the 3′-FAM-labeled blocking oligonucleotide. Additionally, capillary electrophoresis was used for high-throughput and quantitative analysis of reaction products and intermediates with the single-base resolution. To generate the substrate, the 5′-TAM extension primer (50 nm) and 3′-FAM-blocking oligonucleotide (75 nm) were annealed to circular ssM13mp18 DNA (50 nm) in 1× ThermoPol buffer (20 mm Tris-HCl, pH 8.8, at 25 °C, 10 mm (NH_4_)_2_SO_4_, 10 mm KCl, 2 mm MgSO_4_, 0.1% Triton X-100) by heating to 95 °C for 3 min followed by slow cooling to room temperature. The FAM-labeled blocking primer has a modified 3′ end that is not extended by the DNA polymerases in these reactions. It is important to note that the 3′-FAM-labeled blocking primer is in molar excess over the template and extension primer and thus a fraction remains un-annealed and unreacted. In addition, because the TAM fluorescence quantum yield is lower than FAM, an equimolar amount of TAM substrate will have lower fluorescence signal.

Typical Okazaki fragment maturation reactions were performed by mixing Okazaki fragment maturation substrate (5 nm), ATP (1 mm), dNTP (0.1 mm), proliferating cell nuclear antigen (PCNA) (200 nm as a trimer), replication factor C (RFC) (400 nm), polB (10 nm), polD (10 nm), 9°N Fen1 (10 nm), and DNA ligase (10 nm) in 1× ThermoPol buffer. Reactions were incubated at 60 °C for 10 min and terminated with EDTA (100 mm final concentration). All Okazaki fragment maturation reactions were separated by capillary electrophoresis using a 3730xl DNA Analyzer (Applied Biosystems) and fluorescent peaks were analyzed using Peak Scanner software version 1.0 (Applied Biosystems).

**FIGURE 1.**
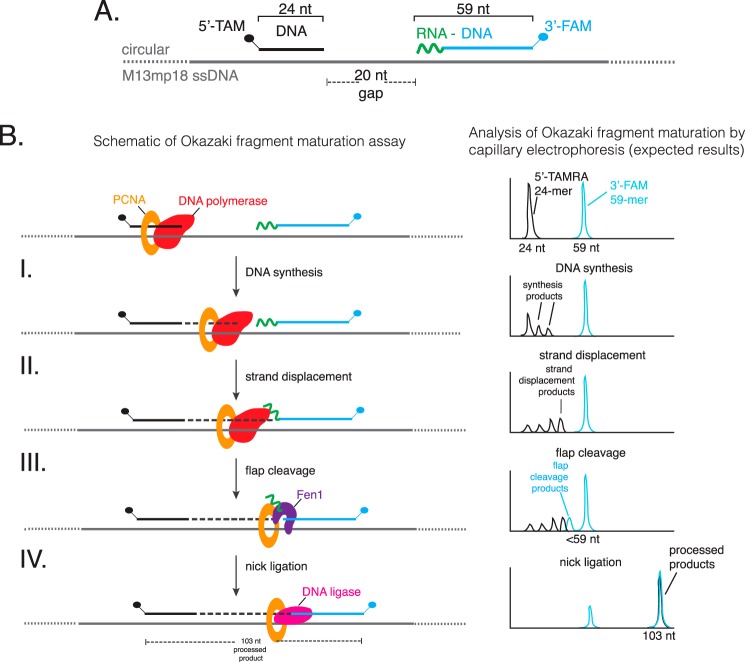
**Dual-label fluorescence assay to monitor Okazaki fragment maturation.** *A,* an Okazaki fragment maturation substrate was prepared by annealing a 5′-TAM-labeled extension primer (24 nt, shaded *black*), a 3′-FAM-labeled blocking oligonucleotide containing 10 nt of 5′ RNA (*green*) followed by 49 nt DNA (*blue*) and circular single-stranded M13mp18 DNA. *B,* a simplified schematic of Okazaki fragment maturation and expected results of capillary electrophoresis. *Panel I*, together with PCNA/RFC, a DNA polymerase initiates synthesis from the 5′-TAM-extension primer resulting in products longer than 24 nt. *Panel* II, DNA polymerase strand displacement of the downstream Okazaki fragment creates flap structures. *Panel* III, Fen1 cleavage products are observed as shorter 3′-FAM-labeled products. *Panel* IV, the remaining DNA:DNA nick is then sealed by DNA ligase to generate a dual 5′-TAM- and 3′-FAM-labeled processed Okazaki fragment (103 nt).

The Okazaki fragment maturation assay is schematically illustrated in Fig. 1. A 5′-TAM-labeled oligonucleotide (*black* in Fig. 1) mimics the lagging strand that is being synthesized. A 3′-FAM-labeled oligonucleotide (*blue* in Fig. 1) having 5′-RNA (*green*) and 3′-DNA (*blue*) regions simulates a previously synthesized, downstream, Okazaki fragment. Because each substrate is labeled with a different fluorescent dye, reaction intermediates and processed products can be analyzed simultaneously by capillary electrophoresis. A schematic of the Okazaki fragment maturation substrate is shown in Fig. 1*A* and expected results of the capillary electrophoresis analysis are depicted in Fig. 1*B*. Synthesis by a DNA polymerase extends the 5′-TAM-labeled primer yielding longer synthesis products (Fig. 1, step I, *black peaks*). Further synthesis extends 5′-TAM-labeled primers and displaces the downstream Okazaki fragment (Fig. 1, steps II and III, *black peaks*) to produce flap structures. Fen1 cleaves the flap structures resulting in shorter 3′-FAM-labeled oligonucleotide (Fig. 1, step III, *blue peaks*). Finally nick ligation joins the extended 5′-TAM-primer and 3′-FAM oligonucleotide as a 103-nt processed product (Fig. 1, step IV, *black* and *blue peaks*).

A control was performed to confirm the expected size of the 103-nt processed product. A 440-nt 5′-TAM-oligonucleotide representing a full-length extension product and a 59-nt 5′-phosphorylated 3′-FAM-labeled blocking oligonucleotide was annealed to single-stranded M13mp18 DNA as described above. The substrate was ligated with T4 DNA ligase in T4 DNA ligation buffer (50 mm Tris-HCl, pH 7.5, 10 mm MgCl_2_, 1 mm ATP, 10 mm DTT) and analyzed by capillary electrophoresis to confirm the presence of the expected 103-nt size product (data not shown).

##### Fen1 and DNA Ligase Activity Assays

A Fen1 DNA substrate was prepared by annealing a 3′-FAM-labeled oligonucleotide (5′-GTT AGT TCG AGC GTA ATG CCC TAT AGT GAG TCG TAT TAA GGT TGT AAA ACG ACG GCC AGT GCC AAG CTT GCA TGC CTG CA-FAM-3′) (50 nm) containing a 40-nt flap, an unlabeled Fen1 primer (5′-CGC CAG GGT TTT CCC AGT CAC GAC G-3′) (75 nm), and ssM13mp18 (75 nm) in 1× ThermoPol buffer. The Fen1 flap DNA substrate (5 nm) was mixed with Tko or immunodepleted extract (2–85 ng) in 1× ThermoPol buffer and incubated for 30 min at 65 °C.

A DNA ligase substrate was prepared by annealing a 5′-FAM labeled oligonucleotide acceptor (5′-FAM-CGC CAG GGT TTT CCC AGT CAC GAC-3′) (50 nm), a 5′-phosphorylated oligonucleotide donor (5′-P-GTT GTA AAA CGA CGG CCA GTG CCA AGC TTG-3′) (75 nm), and single-stranded M13mp18 (75 nm) (ssM13mp18) in 1× ThermoPol supplemented with 1 mm ATP. Tko or immunodepleted extract (0.1–8 μg) was added and the reaction incubated for 30 min at 65 °C. Fen1 and DNA ligase reactions were separated and analyzed by capillary electrophoresis as described above.

## RESULTS

### 

#### 

##### Requirements for Okazaki Fragment Maturation

**FIGURE 2.**
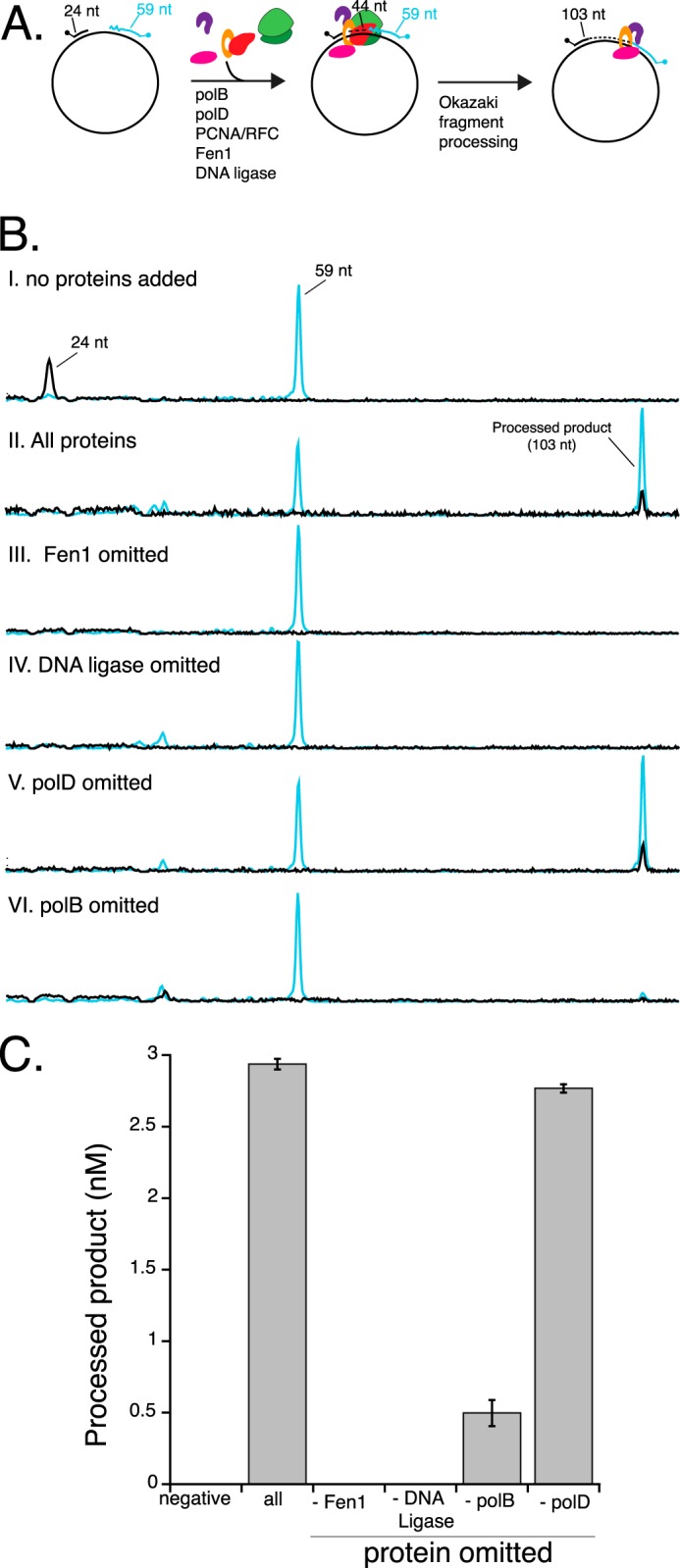
**Determinants of *Thermococcus* Okazaki fragment maturation.** *Panel I*, a schematic of the Okazaki fragment maturation experiment is shown. The processing substrate was prepared by annealing a 5′-TAM-labeled extension primer (shaded *black*) and 3′-FAM-labeled blocking oligonucleotide (shaded *blue*) to ssM13 as described in the text. 9°N polB, polD, PCNA/RFC, Fen1, and DNA ligase were incubated with the processing substrate at 60 °C for 15 min. Processed products (103 nt) result from complete Okazaki fragment maturation. *B*, Okazaki fragment maturation assays were performed without any proteins added, all proteins added (9°N polB, polD, PCNA/RFC, Fen1, and DNA ligase), or in reactions that omit one protein as noted in the figure. Reaction products were analyzed by capillary electrophoresis. 5′-TAM-labeled extension primer is shaded *black* and 3′-FAM-labeled blocking oligonucleotide is shaded *blue*. Processed products (103 nt) result from a complete Okazaki fragment maturation assay and are labeled with both 5′-TAM (*black*) and 3′-FAM (*blue*). *C,* processed products from reactions omitting various replication proteins in *panel B* were quantitated and plotted. The data shown are averages with standard deviations from three independent experiments.

A previous study suggested that in Archaea and Eukarya, a minimal Okazakisome consists of a DNA polymerase, Fen1, DNA ligase, and PCNA (loaded by RFC) (11). Together these proteins efficiently synthesize the individual lagging strand fragments, remove RNA primers, and seal DNA fragments into a contiguous lagging strand. To determine proteins required for Okazaki fragment maturation in *Thermococcus*, processing assays were performed on the dual-labeled DNA construct with either all predicted Okazakisome components present, including polB, polD, PCNA, RFC, Fen1, and DNA ligase, or lacking a particular Okazakisome enzyme (schematically illustrated in Fig. 2*A*). No product could be detected without proteins added to the reaction (Fig. 2*B, panel I*). In reactions containing all components, a 103-nt long product can be observed after 15 min of incubation (Fig. 2*B, panel II*). In the absence of Fen1, DNA polymerase extends the 5′-TAM-labeled extension primer but processing fails due to lack of flap cleavage (Fig. 2*B*, *panel III*). Similarly, without DNA ligase, processing fails because nicks generated by Fen1 cleavage are not sealed (Fig. 2*B, panel IV*). It is important to note that in reactions that lack either Fen1 or DNA ligase, the 5′-TAM-primer is extended beyond the CE analysis view (>800 nt) and is not shown in the figure. Also, in the reaction lacking DNA ligase (Fig. 2*B*, *panel IV*), the blocking oligonucleotide is displaced by DNA polymerase creating a flap that is rapidly degraded by Fen1. Fen1 degradation products are observed at shorter times (data not shown). The remaining 3′-FAM-blocking oligonucleotide shown in Fig. 2*B, panel IV*, is excess unannealed and unreacted oligonucleotide.

**FIGURE 3.**
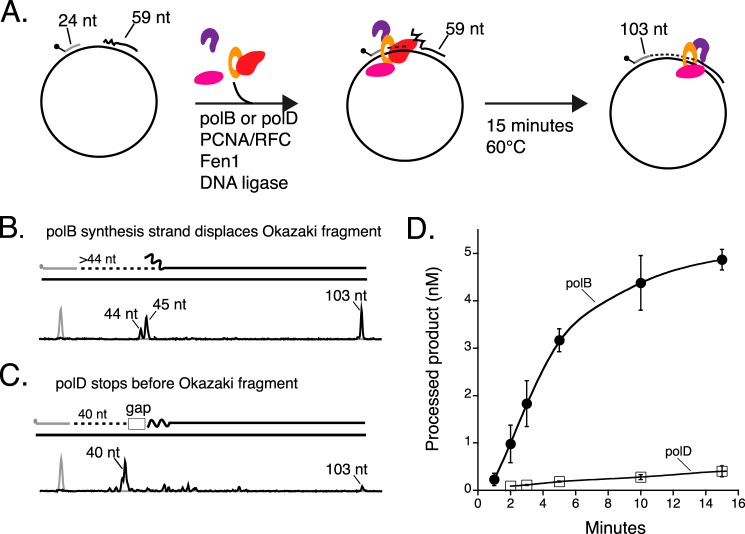
**polB strand displacement is required for efficient Okazaki fragment maturation.** *A,* a schematic of the Okazaki fragment maturation experiment is shown. A DNA polymerase with PCNA/RFC extends a primer. Processed Okazaki fragments result from DNA polymerase strand displacement of the downstream blocking oligonucleotide followed by processing by Fen1 and DNA ligase. Okazaki fragment maturation assays were carried out with either (*B*) polB or (*C*) polD as the DNA polymerase. *B,* polB strand displacement synthesis (15 min incubation) (>44 nt) efficiently creates flap structures that are subsequently processed by Fen1 and DNA ligase (103 nt). *C*, a majority of polD synthesis (15 min incubation) stops upstream of the blocking oligonucleotide (40 nt) leaving a 4-nt gap. A residual amount of polD synthesis continues to generate flaps for Okazaki fragment maturation (103 nt). *D,* quantification of Okazaki fragment maturation. The data shown are averages with standard deviations from three independent experiments with either polB (*filled circles*) or polD (*open squares*).

In reactions containing polB (but lacking polD) Okazaki fragment processing occurs efficiently (Fig. 2*B, panel V*). However, when polB is omitted from the reaction, polD extends the 5′-TAM-primer but little processed product is generated (Fig. 2*B*, *panel VI*). Quantitative values for processed products after 15 min incubation with various Okazakisome proteins is shown in Fig. 3*C*.

##### polD Stops before Encountering a Downstream 5′ Terminus

Full maturation of Okazaki fragments requires displacement of the RNA primer by a DNA polymerase. Thus the ability of polB and polD to perform strand displacement synthesis was monitored by extension of a 5′-TAM-labeled primer annealed to single-stranded M13mp18 template DNA with a downstream blocking oligonucleotide (Fig. 3*A*). Primer extension rates for polB (0.30 min^−1^) and polD (0.45 min^−1^) were similar (data not shown). Extension of the 5′-TAM-primer by a DNA polymerase lacking strand displacement yields a 44-nt fragment. A DNA polymerase with strand displacement activity will synthesize longer products (>44 nt) and displace the downstream blocking oligonucleotide to generate 5′ flap structures (Fig. 3*A*). In the presence of Fen1 and DNA ligase, these flaps are rapidly cleaved with Fen1 and nicks sealed by DNA ligase to generate a 103-nt processed product.

polB synthesis displaces the blocking oligonucleotide resulting in 44- and 45-nt products (Fig. 3*B*). As further polB strand displacement synthesis continues, flaps are rapidly cleaved by Fen1 and nicks are sealed by DNA ligase to generate the 103-nt processed products (Fig. 3, *B* and *D*). In contrast, polD displays weak strand displacement synthesis. The majority of polD synthesis stops upon encountering the downstream Okazaki fragment (40-nt product) leaving a short 4-nt gap (Fig. 3*C*). The stalling of polD 4-nt before downstream fragments was confirmed using a panel of different sized extension primers. The presence or absence of PCNA did not effect polD stalling. The same stalling activity was observed by polD alone or in reactions supplemented with other accessory factors including single-stranded binding proteins or GINS (data not shown). A small fraction (∼5%) of Okazaki fragments are processed to the 103-nt product presumably due to weak polD strand displacement activity that is sufficient for only a trace amount of product formation (Fig. 3, *C* and *D*).

##### polB Completes Okazaki Fragment Maturation After polD Synthesis Stops

**FIGURE 4.**
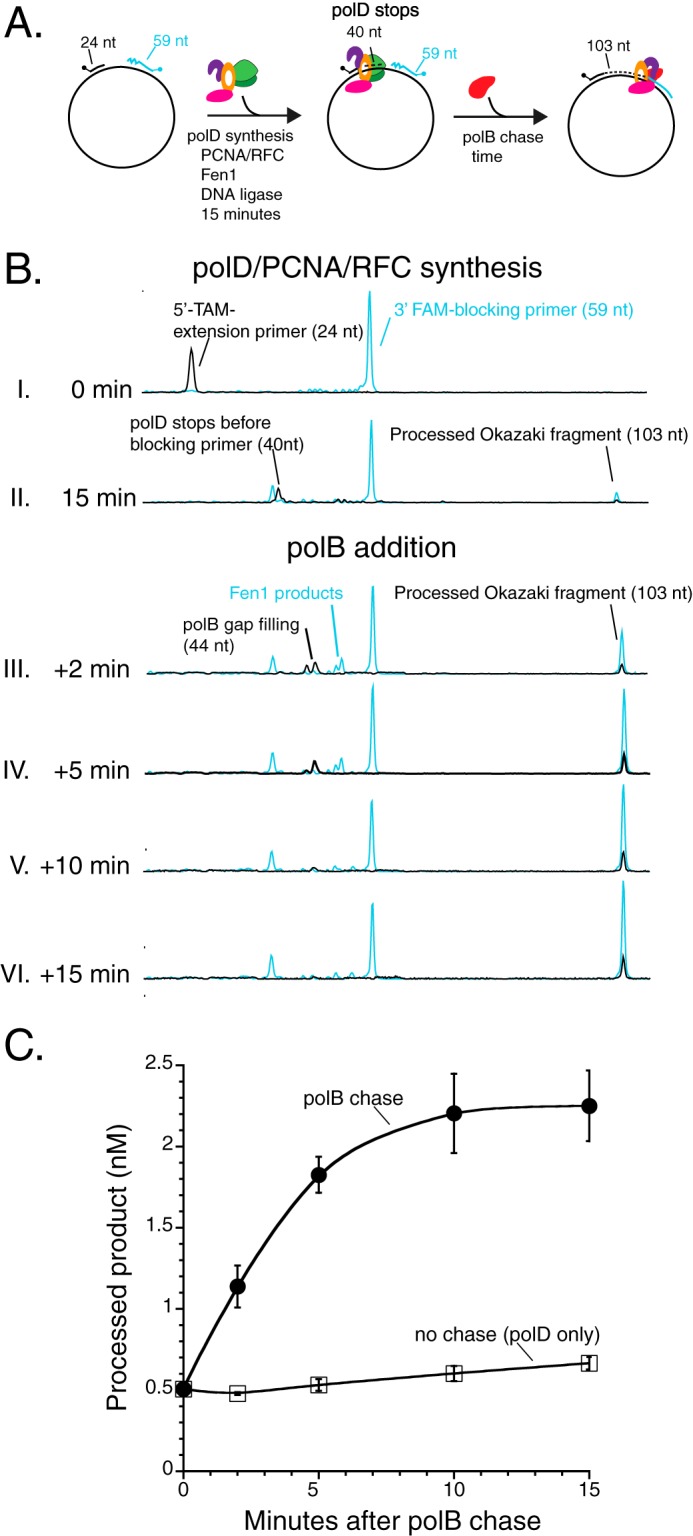
**polB completes Okazaki fragment maturation after polD synthesis stops.** *A,* schematic of Okazaki fragment maturation experiment. polD, PCNA/RFC, Fen1, and DNA ligase were incubated with substrate for 15 min at 60 °C as described under “Experimental Procedures.” After 15 min of incubation, polB was added and reaction aliquots were sampled after 2, 5, 10, and 15 min and halted with 0.25 m EDTA. *B,* reaction products were resolved and analyzed by capillary electrophoresis. DNA polymerase synthesis products are 5′-TAM-labeled (*black*), Fen1 products are 3′-FAM-labeled (*blue*), and Okazaki fragment maturation products are labeled with both 5′-TAM and 3′-FAM (*black* and *blue*). *C,* the data shown are averages with standard deviations from three independent experiments with either polB chase (*filled circles*) or no chase (polD alone, *open squares*).

During lagging strand synthesis by polD, synthesis stops leaving 4-nt gaps before most of the fragments are joined. To complete Okazaki fragment maturation, polB may process gaps left by polD synthesis. To test this hypothesis, polD, PCNA/RFC, Fen1, and DNA ligase were incubated for 15 min at 60 °C in the Okazaki fragment maturation assay. As observed previously, despite detection of a small amount of processed product, the majority of polD synthesis stops upon encountering a downstream Okazaki fragment leaving a 4-nt single-stranded DNA gap (Fig. 4*B, panel II*). Additional incubation (15 min) did not significantly increase the amount of processed product (data not shown).

After the 15-min incubation in the presence of polD with PCNA/RFC, Fen1, and DNA ligase, polB was added and aliquots were sampled over time (Fig. 4, *B, panels III* and VI, and C). polB rapidly fills the single-stranded DNA gap left by polD (Fig. 4*B, panel III*). polB strand displacement synthesis creates flap structures that are processed by Fen1 (Fig. 4*B, panels III* and *IV*). Finally, a nick is sealed by DNA ligase producing a processed 103-nt Okazaki fragment (Fig. 4*B, panels III* and *VI*). Therefore, under these reaction conditions, polB completes Okazaki fragment maturation after stalled polD lagging strand synthesis (Fig. 4*C*).

##### Efficient Okazaki Fragment Requires polB in Tko Cell Extracts

**FIGURE 5.**
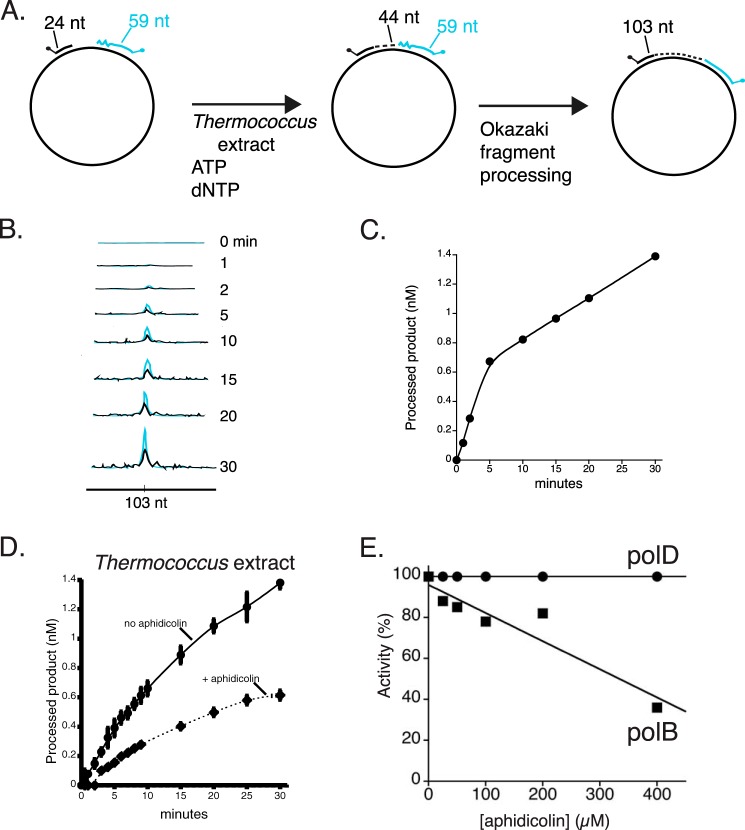
**Okazaki fragment maturation in *Thermococcus* extracts.** *Thermococcus* extract was prepared and incubated with the Okazaki fragment maturation substrate at 60 °C (schematically illustrated in *panel A*). Reaction aliquots were sampled at 1, 5, 10, 15, 20, 25, and 30 min and halted with EDTA. Processed Okazaki fragment products (103 nt) were detected by capillary electrophoresis (*B*) and quantified (*C*). *D,* Tko extract was incubated with the Okazaki fragment maturation substrate at 60 °C in the presence (200 μm; *diamonds*) or absence of aphidicolin (*filled circles*) and analyzed as above. *E,* DNA synthesis by purified polB (10 nm) (*filled squares*) and polD (10 nm) (*filled circles*) was measured in the presence of increasing concentrations of aphidicolin (0–400 μm) and plotted as a percentage of activity in a reaction without aphidicolin.

Tko extracts were prepared and tested for Okazaki fragment maturation (schematically illustrated in Fig. 5*A*). As expected, Okazaki fragments were processed by Tko extracts (Fig. 5, *B* and *C*). In addition, Tko extract processing activity is sensitive to the polB-specific inhibitor aphidicolin (Fig. 5*D*). Because aphidicolin only inhibits polB and not polD (Fig. 5*E*), polB may play a major role in Okazaki processing in cell extracts.

**FIGURE 6.**
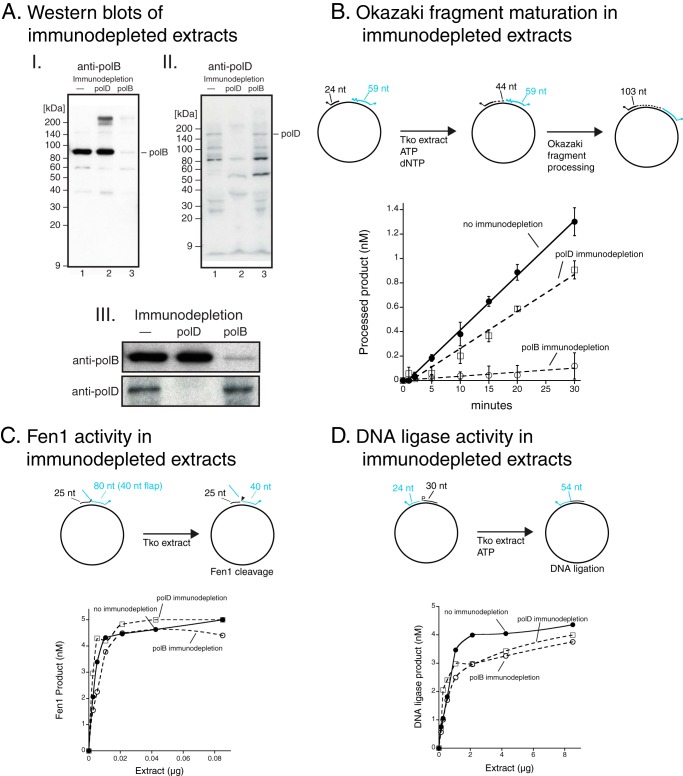
**Okazaki fragment maturation in polB or polD immunodepleted Tko extracts.** Either anti-polB or anti-polD guinea pig antibodies were used to immunodeplete polB or polD from Tko extract. *A,* immunodepleted Tko extracts were separated by electrophoresis, transferred onto membranes, and probed by incubation with rabbit polyclonal antisera raised against polB (*panel I*) or polD (*panel II*). Western blots from *panels I* and *II* were cropped to highlight levels of polB or polD, respectively (*panel III*). Tko extracts without immunodepletion (*filled circles*) or immunodepleted for polB (*open circles*) or polD (*open squares*) were assayed for *B,* Okazaki fragment processing activity; *C,* Fen1 activity; and *D,* DNA ligase activity as described in the text.

Immunodepletion was used to further analyze the roles of polB and polD in Okazaki fragment processing. Immunodepletion has been successfully used in systems that lack genetic tools such as *Xenopus* to dissect replication mechanisms (12). Antibodies raised against either polB or polD were used to immunodeplete each polymerase from the extract. Immunodepletion of polB or polD was confirmed by Western blot (Fig. 6*A*). A Tko extract immunodepleted for polD retains >70% Okazaki fragment processing activity compared with undepleted extracts (Fig. 6*B*). However, maturation is significantly reduced (<6% activity) when polB is immunodepleted (Fig. 6*B*). Other critical activities for Okazaki fragment maturation such as Fen1 and DNA ligase were similar between immunodepleted and non-depleted extracts (Fig. 6, *C* and *D*). Together these data suggest that in cell extracts, the major Okazaki processing DNA polymerase is polB and not polD.

## DISCUSSION

During lagging strand synthesis, short Okazaki fragments are synthesized discontinuously but must be processed together into an uninterrupted lagging strand. The process of joining Okazaki fragments includes the removal of RNA primers at the 5′ end of each fragment, gap filling, and ligating DNA fragments together. In Archaea, Okazaki fragments are short (100–150 nt) and thus over 14,000 Okazaki fragments are synthesized during *Thermococcus* genome replication (13). These fragments must be ligated together rapidly and efficiently to maintain genome integrity. To assess *Thermococcus* species 9°N Okazaki fragment maturation, the process was reconstituted *in vitro* using purified proteins.

It is possible that polB may synthesize the lagging strand in its entirety. However, polB lacks several important characteristics required for a lagging strand polymerase. polB extends RNA primers poorly and lacks interactions with core replisome components (7, 9). In addition, polB is not essential for viability in Tko (5). Thus, polB may play a more specialized role in gap filling and strand displacement synthesis during Okazaki fragment maturation.

On the other hand, previous studies suggest that polD is the major lagging strand polymerase (9, 14, 15). polD fulfills many of the requirements for lagging strand synthesis including efficient RNA primer extension during lagging strand synthesis and interaction with core replisome proteins that couple leading and lagging strand synthesis (9, 14, 15). In addition, polD is the only essential polymerase for viability in Tko and possibly performs both leading and lagging strand synthesis (5). Even though polD is likely the major lagging strand polymerase, this study shows that it cannot efficiently complete Okazaki fragment maturation on its own even in the presence of accessory factors such as single-stranded DNA-binding proteins (RPA), PCNA, and RFC. Data shows that polD synthesis halts before strand displacing the downstream Okazaki fragment, a required activity for processing. Therefore an additional specialized DNA polymerase such as polB likely completes Okazaki fragment maturation by strand displacement synthesis. Data from this study with either purified proteins or with cell extracts support a pivotal role of polB in this process.

**FIGURE 7.**
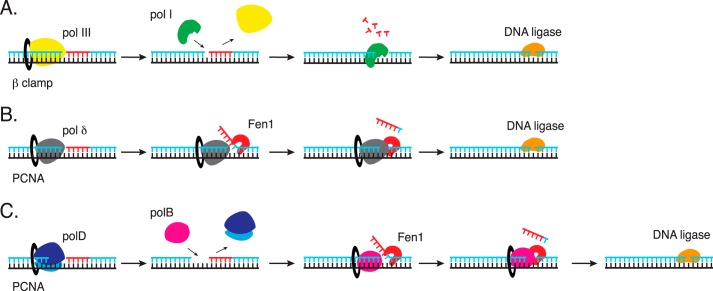
**Simplified models of Okazaki fragment maturation in bacteria (*A*), Eukarya (*B*), and *Thermococcus* species 9°N (*C*).** *A,* in bacteria, pol III synthesizes the lagging strand. pol I replaces pol III to complete Okazaki fragment maturation. pol I 5′-3′ exonuclease removes the RNA primer as its DNA polymerase activity fills the gap. DNA ligase seals the Okazaki fragments. *B,* the eukaryal lagging strand DNA polymerase, pol δ, strand displacement activity generates a flap for Fen1 cleavage. DNA ligase seals the Okazaki fragments. *C,* polD synthesizes the lagging strand and stops at a downstream Okazaki fragment. polB replaces polD and its strand displacement activity generates a flap for Fen1 cleavage. DNA ligase seals the Okazaki fragments. Further details are described in the text.

Based on data from this and previous studies (reviewed in Ref. 10), a more comprehensive model of *Thermococcus* lagging strand processing is proposed (Fig. 7*C*). According to the model, DNA primase synthesizes the primer on the lagging strand. Then polD extends the RNA primer to synthesize the lagging strand but stops 4 nt before the downstream fragment. polB synthesis fills the 4-nt single-stranded gaps and displaces the 5′-end region of the downstream fragment to form a flap structure. Fen1 cleaves the flap and produces a nick for ligation by DNA ligase. It is unknown how polB is directed to the unfinished Okazaki fragment. One possibility may be a direct interaction between polD and polB to hand off synthesis after polD stops. However, polB and polD do not form a stabile protein complex (data not shown) suggesting that other mechanisms or factors may help direct polB to the gapped site.

Thus, the data presented here suggest that *Thermococcus* Okazaki fragment maturation shares elements from both bacterial and eukaryal systems (summarized in Fig. 7). Bacteria and *Thermococcus* utilize separate DNA polymerases for lagging strand synthesis and Okazaki fragment maturation. Like bacteria, *Thermococcus* requires another DNA polymerase (pol I or polB, respectively) for efficient Okazaki fragment maturation (Fig. 7) (16). In contrast to bacteria where pol I performs both primer removal and gap filling, *Thermococcus* polB lacks 5′-3′ exonuclease activity and does not remove the primer itself. Similar to eukaryotic pol δ, strand displacement activity of polB forms a flap structure that is cleaved by Fen1 (2). In all three domains, DNA ligase seals the nicks.

In addition to functioning in Okazaki fragment maturation, polB gap filling and strand displacement synthesis may also play important roles during DNA repair, recombination, and replication fork restart. The increased UV sensitivity of Tko strains lacking polB suggests an important role for polB in DNA nucleotide excision repair (5). In addition, *Escherichia coli* polB has been shown to be important for replication restart after replisome stalling at lesions and archaeal polB uracil recognition may be a mechanism to recruit DNA repair factors (17, 18). Together, these data suggests that polB gap filling and strand displacement synthesis *in vivo* could play multiple roles in Okazaki fragment maturation, DNA repair, recombination, and replication restart.

Lagging strand synthesis is highly dynamic and during synthesis a signal must trigger the lagging strand polymerase to switch from synthesis mode to the next Okazaki fragment (16). In *Thermococcus*, mechanisms for lagging strand loop release and polymerase recycling are not understood. However, data from this study suggests that polD stops before the 5′ terminus and may be a signal to trigger polymerase recycling to a new RNA primer. Alternatively, other factors such as DNA primase primer synthesis may signal polD to release the replication loop before the Okazaki fragment is completely synthesized. Further investigations are required to determine whether the signaling or collision (or both) mechanism occurs within the archaeal replisome during coordinated DNA replication.

It is not clear if all archaeal species use similar mechanisms for Okazaki fragment maturation. The genomes of organisms belonging to the crenarchaeota branch do not encode a polD homologue (3). However, all known crenarchaea encode for at least two homologues of polB (19) (17). It is possible that one crenarchaeal polB homolog performs both leading and lagging synthesis, whereas the other homolog completes Okazaki fragment maturation. Alternatively, it is possible that a single crenarchaeal polB is involved in strand replication and Okazaki fragment maturation, whereas the other participates in other cellular processes such as recombination or DNA repair. Future studies are needed to address this question.

This study shows that polB is pivotal for Okazaki fragment maturation in *Thermococcus.* Further studies will illuminate if other archaeal kingdoms also process Okazaki fragments in a similar fashion. For example, in some Archaea polB is essential for viability (6), whereas it is not essential in others (4, 5). This suggests that other enzymes may substitute for polB activities in those organisms in which polB is deleted. It is possible that in these species, polD has a stronger strand displacement activity or that the low strand displacement activity by polD or DNA primase (data not shown) may be sufficient to support Okazaki fragment maturation. Archaea encode for RNase H2 homologs that may serve as a redundant pathway for RNA primer removal in place of Fen1 (14). It was also suggested that RNA primers may be removed by an exonuclease that is a part of the replisome (20). Additional study is required to understand variations in Okazaki fragment maturation mechanisms among the Archaea.

## Footnotes

2 The abbreviations used are:

TAM: 5-carboxytetramethylrhodamine
FAM: 5-carboxyfluorescein
Fen1: flap endonuclease 1
PCNA: proliferating cell nuclear antigen
RFC: replication factor C
nt: nucleotide.
